# Graph convolutional network-based feature selection for high-dimensional and low-sample size data

**DOI:** 10.1093/bioinformatics/btad135

**Published:** 2023-04-21

**Authors:** Can Chen, Scott T Weiss, Yang-Yu Liu

**Affiliations:** Channing Division of Network Medicine, Department of Medicine, Brigham and Women’s Hospital, Harvard Medical School, Boston, MA 02115, United States; Channing Division of Network Medicine, Department of Medicine, Brigham and Women’s Hospital, Harvard Medical School, Boston, MA 02115, United States; Channing Division of Network Medicine, Department of Medicine, Brigham and Women’s Hospital, Harvard Medical School, Boston, MA 02115, United States; Center for Artificial Intelligence and Modeling, The Carl R. Woese Institute for Genomic Biology, University of Illinois at Urbana-Champaign, Champaign, IL 61820, United States

## Abstract

**Motivation:**

Feature selection is a powerful dimension reduction technique which selects a subset of relevant features for model construction. Numerous feature selection methods have been proposed, but most of them fail under the high-dimensional and low-sample size (HDLSS) setting due to the challenge of overfitting.

**Results:**

We present a deep learning-based method—GRAph Convolutional nEtwork feature Selector (GRACES)—to select important features for HDLSS data. GRACES exploits latent relations between samples with various overfitting-reducing techniques to iteratively find a set of optimal features which gives rise to the greatest decreases in the optimization loss. We demonstrate that GRACES significantly outperforms other feature selection methods on both synthetic and real-world datasets.

**Availability and implementation:**

The source code is publicly available at https://github.com/canc1993/graces.

## 1 Introduction

Many biological data representations are naturally high-dimensional and low-sample size (HDLSS) (Berrar et al. 2003; Leung and Cavalieri 2003; Alipanahi et al. 2015; Auton et al. 2015; Uffelmann et al. 2021). RNA sequencing (RNA-Seq) is a next-generation sequencing technique to reveal the presence and quantity of RNA in a biological sample at a given moment (Kukurba and Montgomery 2015). RNA-Seq datasets often contain a huge amount of features (e.g. $\geq 10^{5}$), while the number of samples is very small (e.g. $\leq 10^{3}$). Analyzing RNA-Seq data is crucial for various disciplines in biomedical sciences, such as disease diagnosis and drug development (Berrar et al. 2003; Leung and Cavalieri 2003). However, such data not only have low-sample sizes, but its features might also be highly collinear (i.e. linearly correlated). Both attributes would lead to the challenge of overfitting, i.e. poor generalizability, when performing machine learning tasks such as feature selection on HDLSS data (Kim and Kim 2018).

A useful technique in dealing with high-dimensional data is feature selection, which aims to select an optimal subset of features. Although the selection of an optimal subset of features is an NP-hard problem (Chen et al. 1997), various compromised feature selection methods have been proposed. While feature selection methods are often grouped into filtering, wrapped, and embedded methods (Stańczyk 2015), in this article, we classify them into five categories—statistics-based (Golugula et al. 2011; Zuber and Strimmer 2011; Bommert et al. 2020), Lasso-based (Tibshirani 1996; Yamada et al. 2014), decision tree-based (Xu et al. 2014; Bommert et al. 2020), deep learning-based (Li et al. 2016; Liu et al. 2017), and greedy methods (Aha and Bankert 1995), according to their learning schemes, see details in Section 2. Note that most of the methods address the curse of dimensionality under the blessing of large-sample size (Liu et al. 2017). Only a few of them can handle HDLSS data. The state-of-the-art feature selection methods for HDLSS data are Hilbert–Schmidt independence criterion (HSIC) Lasso (Yamada et al. 2014, 2018) and deep neural pursuit (DNP) (Liu et al. 2017).

In this article, we propose a graph neural network-based feature selection method—GRAph Convolutional nEtwork feature Selector (GRACES)—to extract features by exploiting the latent relations between samples for HDLSS data. Inspired by DNP, GRACES is a deep learning-based method that iteratively finds a set of optimal features. GRACES utilizes various overfitting-reducing techniques, including multiple dropouts, introduction of Gaussian noises, and F-correction, to ensure the robustness of feature selection. We demonstrate that GRACES outperforms HSIC Lasso and DNP (and other baseline methods) on both synthetic and real-world datasets.

The article is organized into six sections. We perform a thorough literature review on feature selection (including traditional and HDLSS feature selection methods) in Section 2. The main architecture of GRACES is presented in Section 3. We evaluate the performance of GRACES along with several representative methods on both synthetic and real-world datasets in Section 4. We perform an ablation study and discuss the drawbacks of GRACES in Section 5. Finally, we conclude with future research directions in Section 6.

## 2 Related work

Univariate statistical tests have been widely applied for feature selection (Bommert et al. 2020; Golugula et al. 2011). The computational advantage allows them to perform feature selection on extremely high-dimensional data. The ANOVA (analysis of variance) F-test (Stahle et al. 1989) is one of the most commonly used statistical methods for feature selection. The value of the F-statistic is used as a ranking score for each feature, where the higher the F-statistic, the more important is the corresponding feature (Bommert et al. 2020). Other classical statistical methods, including the student’s t-test (Owen 1965), the Pearson correlation test (Meng et al. 1992), the Chi-squared test (Plackett 1983), the Kolmogorov–Smirnov test (Daniel 1990), the Wilks’ lambda test (El Ouardighi et al. 2007), and the Wilcoxon signed-rank test (Wilcoxon 1992), can be applied for feature selection in a similar manner. Empirically, the ANOVA F-test is able to achieve a relatively good performance in feature selection on some HDLSS data with very low computational costs. Besides statistical tests, other tools such as correlation-adjusted correlation estimation/regression (Zuber and Strimmer 2011) and Bayesian analysis (Krishnapuram et al. 2004; Constantinopoulos et al. 2006; Feng et al. 2012) have been used for feature selection.

L1-regularization, also known as the least absolute shrinkage and selection operator (Lasso), has a powerful built-in feature selection capability for HDLSS data (Tibshirani 1996). Lasso assumes linear dependency between input features and outputs, penalizing on the *l*_1_-norm of feature weights. Lasso produces a sparse solution with which the weights of irrelevant features are zero. Yet, Lasso fails to capture nonlinear dependency. Therefore, kernel-based Lasso such as HSIC Lasso (Yamada et al. 2014, 2018) has been developed for handling nonlinear feature selection on HDLSS data. HSIC Lasso utilizes the empirical HSIC (Gretton et al. 2005) to find non-redundant features with strong dependence on outputs. HSIC Lasso outperforms other similar methods, including feature vector machine (Li et al. 2005), minimum redundancy maximum relevance (Peng et al. 2005), sparse additive model (Ravikumar et al. 2009), quadratic programming feature selection (Rodriguez-Lujan et al. 2010), and centered kernel target alignment (Cortes et al. 2012). Additionally, the *l*_1_-regularizer in Lasso can be compatibly incorporated into different classifiers such as logistic regression (LR Lasso) for feature selection (Meier et al. 2008).

Decision tree-based methods are also popular for feature selection, which can model nonlinear input–output relations (Bommert et al. 2020). As an ensemble of decision trees, random forests (RF) (Breiman 2001) calculates the importance of a feature based on its ability to increase the pureness of the leaf in each tree. A higher increment in leaves’ purity indicates higher importance of the feature. In addition, gradient-boosted feature selection (GBFS) selects features by penalizing the usage of features that are not used in the construction of each tree (Xu et al. 2014). However, decision tree-based feature selection methods such as RF and GBFS require large-sample size for training. Hence, these methods often do not perform well under the HDLSS setting.

Numerous deep learning-based methods have been proposed for feature selection (Li et al. 2016; Chen et al. 2017; Shrikumar et al. 2017; Lu et al. 2018; Borisov et al. 2019; Gui et al. 2019; Mirzaei et al. 2020; Wojtas and Chen 2020). Like decision tree-based methods, deep neural networks also require a large number of samples for training, so these methods often fail on HDLSS data. Nevertheless, there are several deep learning-based feature selection methods which are designed specifically for HDLSS data (Liu et al. 2017; Li et al. 2022). DNP learns features by using a multilayer perceptron (MLP) and incrementally adds them through multiple dropout technique in a nonlinear way (Liu et al. 2017). DNP overcomes the issue of overfitting resulting from low-sample size and outperforms other methods such as LR Lasso, HSIC Lasso, and GBFS on HDLSS data. An alternative to DNP with replacing the MLP by a recurrent neural network is mentioned in (Chowdhury et al. 2019). Yet, DNP only uses MLP to generate low-dimensional representations, which fails to capture the complex latent relationships between samples. Moreover, Deep feature screening incorporates a neural network for learning low-dimensional representations and a multivariate rank distance correlation measure (applied on the low-dimensional representations) for feature screening (Li et al. 2022). However, the effectiveness of the method needs further investigation.

Other frequently used feature selection methods include recursive feature elimination (Guyon et al. 2002) and sequential feature selection (Aha and Bankert 1995). The former recursively considers smaller and smaller sets of features based on the feature importance obtained by training a classifier. The latter is a greedy algorithm that adds (forward selection) or removes (backward selection) features based on the cross-validation score of a classifier. However, both methods are computational expensive, which become infeasible when dealing with HDLSS data.

## 3 Materials and methods

**Figure 1:**
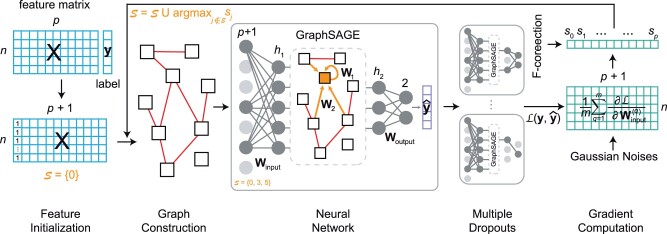
Workflow of GRACES. GRACES consists of feature initialization that adds a bias feature served as the initial selected feature, graph construction with using cosine similarity on the selected features, three-layer neural network with an input linear layer, GraphSAGE layer, and an output linear layer (where gray disks represent the hidden neurons in the neural network), multiple dropouts on the hidden neurons for reducing variance in the subsequent computation, and gradient computation (with introduction of Gaussian noises or F-correction) which gives rise to the current optimal feature according to the gradient magnitude. Note that the activation of the hidden neurons in the input linear layer depends on S

GRACES is an iterative algorithm which has five major components: feature initialization, graph construction, neural network, multiple dropouts, and gradient computation (Fig. 1). Motivated by DNP, GRACES aims to iteratively find a set of optimal features which gives rise to the greatest decreases in the optimization loss. For feature initialization, given a feature matrix $X\in R^{n\times p}$ with n≪p, we first introduce a bias feature (e.g. an all-one column) into **X** and index it by zero. The total number of features now is *p *+* *1, and the original features have the same index numbers as before. We initialize the selected feature set S={0}, i.e. the bias feature. In other words, the bias feature serves as the initial selected feature to start the feature selection process.

For graph construction, we exploit the cosine similarity measure based on the selected features in S. Given two feature vectors $x_{i}\in R^{\left|S\right|}$ and $x_{j}\in R^{\left|S\right|}$ for sample *i* and *j*, the cosine similarity is defined as the cosine of the angle between them in the Euclidean space, i.e.


(1) $$S_{C}(x_{i},x_{j})=\frac{x_{i}^{⊤}x_{j}}{||x_{i}|{|}_{2}||x_{j}|{|}_{2}}.$$

Considering each sample as a node, we connect two nodes if their cosine similarity score is larger than a threshold *δ* (which is a hyperparameter of GRACES). The resulting similarity graph captures the latent interactions between samples and will be used in the graph convolutional network (GCN) layer. The similarity graph is different at each iteration, and other similarity measures, such as Pearson correlation and Chi-squared distance (Wang et al. 2014) (for discrete features), can also be used here.

We build the neural network with three layers: an input linear layer, a GCN layer, and an output linear layer. In order to select the features iteratively, we only need to consider weights along the dimensions corresponding to the selected features in the input weight matrix (in other words, for those non-selected features, the corresponding entries in the weight matrix must be zeros) without a bias vector, i.e. where $x_{j}\in R^{p+1}$ is the feature vector for sample *j*, $W_{\text{input}}\in R^{h_{1}\times (p+1)}$ is the learnable weight matrix (*h*_1_ denotes the first hidden dimension) such that the (i+1)th column is a zero vector for i∉S. Subsequently, we utilize one of the classical GCNs—GraphSAGE (Hamilton et al. 2017) to refine the embeddings based on the similarity graph constructed from the second step, i.e. where $W_{1}\in R^{h_{2}\times h_{1}}$ and $W_{2}\in R^{h_{2}\times h_{1}}$ are two learnable weight matrices (*h*_2_ denotes the second hidden dimension), and N(j) denotes the neighborhood set of node *j*. GraphSAGE leverages node feature information to efficiently generate embeddings by sampling and aggregating features from a node’s local neighborhood (Hamilton et al. 2017). Finally, the refined embedding is further fed into an output linear layer to produce probabilistic scores of different classes for each sample, i.e. where $W_{\text{output}}\in R^{h_{2}\times 2}$ is a learnable weight matrix (assuming the labels are binary, i.e. label zero and label one) and $b_{\text{output}}\in R^{2}$ is the bias vector. We denote the predicted vector containing the probabilities of label one (second entry in ${\hat{y}}_{j}$) for all samples by $\hat{y}\in R^{n}$.


(2) $${\hat{x}}_{j}=\text{ReLU}(W_{\text{input}}x_{j}),$$

(3) $${\tilde{x}}_{j}=\text{ReLU}(W_{1}{\hat{x}}_{j}+\frac{1}{\left|N(j)\right|}\sum_{i\in N(j)}W_{2}{\hat{x}}_{i}),$$

(4) $${\hat{y}}_{j}=\text{Softmax}(W_{\text{output}}{\tilde{x}}_{j}+b_{\text{output}}),$$

To reduce the effect of high variance in the subsequent gradient computation, we adopt the same strategy of multiple dropouts as proposed in Liu et al. (2017). After training the neural network based on the selected features, we randomly drop hidden neurons in the GCN layer and the output layer *m* times with dropout probability *P* (*m* and *P* are hyperparameters of GRACES). In other words, we obtain multiple different dropout neural network models. The technique of multiple dropouts has proved to be effectively stable and robust for deep learning-based feature selection under the HDLSS setting (Liu et al. 2017; Chowdhury et al. 2019).

For gradient computation, we compute the gradient regarding the input weight for each dropout neural network model and take the mean, i.e.

(5) $$G=\frac{1}{m}\sum_{q=1}^{m}\frac{\partial L}{\partial W_{\text{input}}^{\left(q\right)}}\in R^{h_{1}\times (p+1)}$$

where L is the optimization loss, and $W_{\text{input}}^{\left(q\right)}$ is the input weight matrix for the *q*th dropout model. Here we use the cross-entropy loss, i.e.

$$L(y,\hat{y})=-\frac{1}{n}\sum_{j=1}^{n}y_{j}\;\text{log}\;{\hat{y}}_{j}+(1-y_{j})\;\text{log}(1-{\hat{y}}_{j}),$$

where *y_j_* and ${\hat{y}}_{j}$ are the *j*th entries of **y** and $\hat{y}$, representing the true label and the predicted probability of label one for sample *j*, respectively. After obtaining the average gradient matrix, the next selected feature can be computed based on the magnitude of the column norm of **G**, i.e.

(6) $$S=S\cup {\text{argmax}}_{j\notin S}||g_{j}|{|}_{2},$$

where $g_{j}$ is the *j*th column of **G**. The selected feature set is iteratively updated until reaching the number of requested features, and the final features selected by GRACES is given by S with the bias feature removed.

Algorithm 1 GRACES

1: **Input:** Feature matrix $X\in R^{n\times p}$, label vector $y\in R^{n}$, the number of requested feature *K*, score threshold *δ*, hidden dimensions *h*_1_ and *h*_2_, learning rate *l*, number of dropouts *m*, dropout probability *P*, Gaussian variance $\sigma^{2}$, and correction rate *α*

2: Introduce a bias feature into **X** and index it by 0

3: Initialize S={0}

4: **while**|S|≤K+1**do**

5:  Construct a cosine similarity graph based on S with a similarity score threhold *δ*

6:  Train a neural network on **X** and **y** with learning rate *l*, including an input layer (with $W_{\text{input}}\in R^{h_{1}\times (p+1)}$), a GCN layer (with $W_{1},W_{2}\in R^{h_{2}\times h_{1}}$), and an output layer (with $W_{\text{ouput}}\in R^{h_{2}\times 2}$ and $b_{\text{output}}\in R^{2}$)

7:  Dropout *m* times in the GCN and output layers of the neural network with dropout probability *P*

8:  Introduce Gaussian noises (generated from a Gaussian distribution with mean zero and variance $\sigma^{2}$) to the GCN layer

9:  Compute the average gradient regarding the input weight matrix

10:  Correct the feature scores by the ANOVA F-test with correction rate *α*

11:  Update the selected feature set by (8)

12: **end while**

13: Drop the bias feature (i.e. the first element) from S

14: **Return:** Selected feature set S.

To further reduce the effect of overfitting due to low-sample size, we incorporate two additional strategies in GRACES. First, we consider introducing Gaussian noises to the weight matrices of the GCN layer, i.e. adding noise matrices generated from a Gaussian distribution with mean zero and variance $\sigma^{2}$ (which is a hyperparameter of GRACES) to $W_{1}^{\left(q\right)}$ and $W_{2}^{\left(q\right)}$, for the different dropout models in the gradient computation step. Studies have shown that introduction of Gaussian noises is able to boost the stability and the robustness of deep neural networks during training (Yin et al. 2015; Li and Liu 2020; Jang et al. 2021). Second, we consider correcting the feature scores (i.e. $||g_{j}|{|}_{2}$) by incorporating it with the ANOVA F-test, i.e. the final score for feature *j* is given by

(7) $$s_{j}=\alpha g_{j}+(1-\alpha )f_{j},$$

where *g_j_* is the normalized score computed from $||g_{j}|{|}_{2}$, *f_j_* is the normalized score computed from the F-statistic, and α∈[0,1] is the correction weight (which is a hyperparameter of GRACES). Therefore, the selected feature set is updated by the follows:

(8) $$S=S\cup {\text{argmax}}_{j\notin S}s_{j}.$$

The reasons we select the ANOVA F-test are: (i) it is computationally efficient; (ii) it achieves a relatively good performance in feature selection for some HDLSS data; (iii) it does not suffer from overfitting, so including it can reduce the effect of overfitting in GRACES. Other statistical tests, such as the Student’s *t*-test, the Pearson correlation test, and the Wilcoxon signed-rank test, can be applied similarly. More advanced methods like HSIC Lasso or DNP can also be considered, but might require more computational recourses. The two overfitting-reducing strategies effectively improve the performance of GRACES for HDLSS data, see Section 5.

Detailed steps of GRACES can be found in Algorithm 1. We list all the hyperparameters of GRACES in Table 1. Although GRACES is inspired from DNP, it differs from DNP in the following aspects: (i) GRACES constructs a dynamic similarity graph based on the selected feature at each iteration; (ii) GRACES exploits advanced GCN (i.e. GraphSAGE) to refine sample embeddings according to the similarity graph, while DNP only uses MLP which fails to capture latent associations between samples; (iii) in addition to multiple dropouts proposed in DNP, GRACES utilizes more overfitting-reducing strategies, including introduction of Gaussian noises and F-correction, to further improve the robustness of feature selection. In the following section, we will see that GRACES significantly outperforms DNP in both synthetic and real-world examples.

**Table 1:** Hyperparameters of GRACES and their values or search ranges used in the synthetic and real-world data tests.

Hyperparameter	Notation	Synthetic data	Real-world data
Number of requested feature	*K*	10	{1, 2, …, 20}
Similarity score threshold	*δ*	0.95	0.95
First hidden dimension	*h*_1_	64	64
Second hidden dimension	*h*_2_	32	32
Learning rate	*l*	0.001	0.001
Number of dropout	*m*	10	10
Dropout probability	*P*	{0.1, 0.25, 0.75}	{0.1, 0.25, 0.75}
Gaussian variance	$\sigma^{2}$	{0, 0.1, 0.5}	0
Correction rate	*α*	0	{0, 0.1, 0.5, 0.9}

## 4 Experiments

We evaluated the performance of GRACES on both synthetic and real-world HDLSS datasets along with six representative feature selection methods, including the ANOVA F-test (Stahle et al. 1989), LR Lasso (Meier et al. 2008), HSIC Lasso (https://github.com/riken-aip/pyHSICLasso) (Yamada et al. 2014), RF (Breiman 2001), CancelOut (https://github.com/unnir/CancelOut) (a traditional deep learning-based feature selection method) (Borisov et al. 2019), and DNP (https://github.com/KaixuYang/ENNS) (Liu et al. 2017). HSIC Lasso and DNP are recognized as the state-of-the-art methods for HDLSS feature selection. The reason we chose CancelOut is that it achieves a relatively better performance compared to other deep learning-based methods (which are not designed specifically for HDLSS data). We did not compare with GBFS (due to the feature of early stopping), deep feature screening (due to lack of code availability), and recursive feature elimination and sequential feature selection (due to infeasible computation). We used support vector machine as the final classifier and the area under the receiver operating characteristic curve (AUROC) as the evaluation metric for all the methods. All the experiments presented were performed on a Macintosh machine with 32 GB RAM and an Apple M1 Pro chip in Python 3.9.

### 4.1 Synthetic datasets

We used the scikit-learn function make_classification to generate synthetic data. The function creates clusters of points normally distributed about vertices of a *q*-dimensional hypercube (*q* is the number of important features) and assigns an equal number of clusters to each class (Guyon et al. 2004). We set the number of samples to 60 and fixed the number of important features to 10. We varied the total number of features from 500 to 5000 and considered three synthetic datasets with easy, intermediate, and hard classification difficulty (can be controlled by the variable class_sep). We randomly split each dataset into 70% training, 20% validation, and 10% testing with 20 replicates. We performed grid search for finding the optimal key hyperparameters for each method. We reported the average test AUROC (over 20 times train test splits) with respect to the total number of features. In the meantime, since we know the exact important features, we also reported the correction rate of the selected features during training.

**Figure 2:**
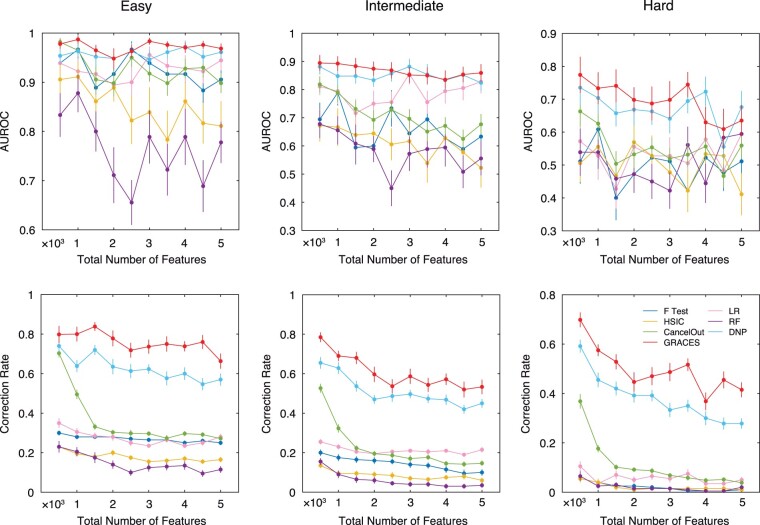
Synthetic datasets. Average test AUROC and correction rate with respect to the total number of features for the easy, intermediate, and hard synthetic datasets. Error bars indicate standard error mean

The results are shown in Fig. 2. Clearly, GRACES achieves a superb performance under all three modes. Notably, GRACES is able to capture more correct important features (i.e. the correction rate of GRACES significantly outperforms the other methods), which leads to a better test AUROC. Moreover, the performance of GRACES is remarkably stable regarding the increase of the total number of features (especially under the easy and intermediate modes). In contrast, the AUROC of the other methods (except DNP) fluctuates drastically. Under the easy mode, most of the methods (such as the ANOVA F-test, LR Lasso, and CancelOut) accomplish a comparable performance (i.e. AUROC > 90%) even though their correction rates are much lower than that of GRACES. Under the hard mode, however, these methods become ineffective (i.e. AUROC ∼50%). Finally, DNP achieves the second-best performance for the three synthetic datasets.

### 4.2 Real datasets

We used the same biological datasets from the DNP paper (Liu et al. 2017), which includes:

- Colon: Gene expression data from colon tumor patients and normal control;
- Leukemia: Gene expression data from acute lymphoblastic leukemia (ALL) patients and normal control;
- ALLAML: Gene expression data from acute lymphoblastic leukemia (ALL) patients and acute myeloid leukemia (AML) patients;
- GLI_85: Gene expression data from glioma tumor patients and normal control;
- Prostate_GE: Gene expression data from prostate cancer patients and normal control;
- SMK_CAN_187: Gene expression data from smokers with lung cancer and smokers without lung cancer.

The statistics of the datasets are shown in Table 2.

**Table 2:** Statistics of the real-world datasets.^a^

Dataset	Colon	Leukemia	ALLAML	GLI_85	Prost._GE	SMK._187
No. of samples	62 (40, 22)	72 (47, 25)	72 (47, 25)	85 (26, 59)	102 (50, 52)	187 (90, 97)
No. of features	2000	7070	7129	22 283	5966	19 993
No. of classes	2	2	2	2	2	2

a The numbers of case and control samples are shown in parentheses.

**Figure 3:**
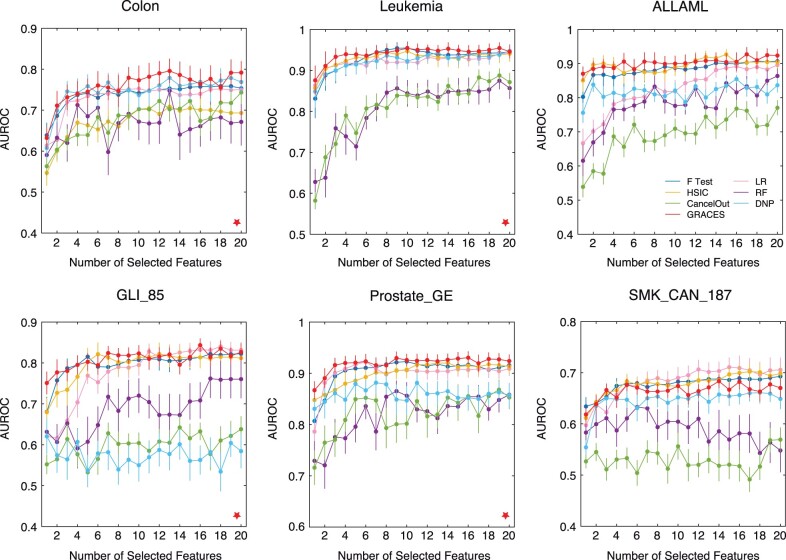
Real-world datasets. Average test AUROC with respect to the number of selected features for each dataset. Error bars indicate standard error mean, and red stars indicate statistical significance compared to the second-best method (*P*-value < .05, one-sample paired *t*-test on the total 400 AUROC scores)

**Figure 4:**
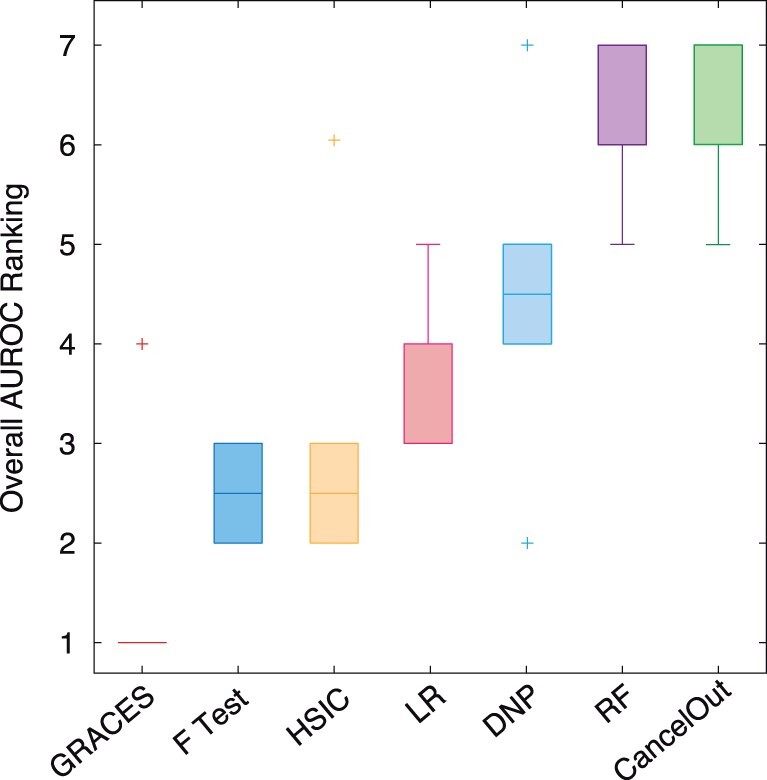
Boxplot of overall AUROC mean ranking over the six datasets for all the feature selection methods. For each dataset, the ranking of a method was determined by the mean of the total 400 AUROC scores as shown in Table 3

We randomly split each dataset into 20% training, 50% validation, and 30% testing with 20 replicates. We chose a such low-training size is that a high-training size would result in an extremely high performance for every method (which can be seen in the DNP paper; Liu et al. 2017). We performed grid search for finding the optimal key hyperparameters for each method. We reported the average test AUROC (over 20 times train test splits) with respect to the number of selected features from 1 to 20. The results are shown in Fig. 3, where GRACES outperforms the other methods for all the datasets except SMK_CAN_187. In particular, on the Colon, Leukemia, GLI_85, and Prostate_GE datasets, the advantage of GRACES can be shown with statistical significance compared to the second-best method (*P*-value < .05, one-sample paired t-test on the total 400 AUROC scores). Moreover, the performance of GRACES is stable and robust across all the datasets, while the other methods (such as LR Lasso, HSIC Lasso, and DNP) would fail on certain datasets (e.g. LR Lasso on ALLAML; HSIC Lasso on Colon; DNP on GLI_85), see Table 3 and Fig. 4. By combining all the AUROC scores obtained from the six datasets, the overall performance of GRACES is significantly better than these of all the other methods (p-value < $10^{-9}$, one-sample paired *t*-test on the total 2400 AUROC scores). Surprisingly, the ANOVA F-test achieves a relative good and stable performance on the real-world datasets. RF and CancelOut, which are not suitable for HDLSS data, do not perform well. We further repeated the experiment with MLP and *k*-nearest neighbors as the final classifiers and observed a similar result, where GRACES achieves a comparable or improved performance over the baselines on the six biological datasets (Supplementary Figs S1 and S2).

**Table 3:** Overall AUROC mean and rankings of the feature selection methods.^a^

Dataset	Colon	Leukemia	ALLAML	GLI_85	Prost._GE	SMK._187
F-test	0.7385 (3)	0.9303 (2)	0.8830 (3)	0.7982 (2)	0.9043 (2)	0.6771 (3)
LR Lasso	0.7308 (4)	0.9218 (5)	0.8249 (4)	0.7778 (4)	0.8997 (4)	**0.6824 (1)**
HSIC Lasso	0.6739 (6)	0.9293 (3)	0.8951 (2)	0.7954 (3)	0.9003 (3)	0.6786 (2)
RF	0.6658 (7)	0.8060 (7)	0.7818 (6)	0.6875 (5)	0.8188 (7)	0.5899 (6)
CancelOut	0.6782 (5)	0.8098 (6)	0.6916 (7)	0.5989 (6)	0.8215 (6)	0.5288 (7)
DNP	0.7474 (2)	0.9234 (4)	0.8173 (5)	0.5740 (7)	0.8594 (5)	0.6454 (5)
GRACES	**0.7591 (1)**	**0.9411 (1)**	**0.9025 (1)**	**0.8089 (1)**	**0.9191 (1)**	0.6644 (4)

a We ranked these methods based on the overall AUROC mean. The results for GRACES were highlighted in bold.

## 5 Discussion

**Figure 5:**
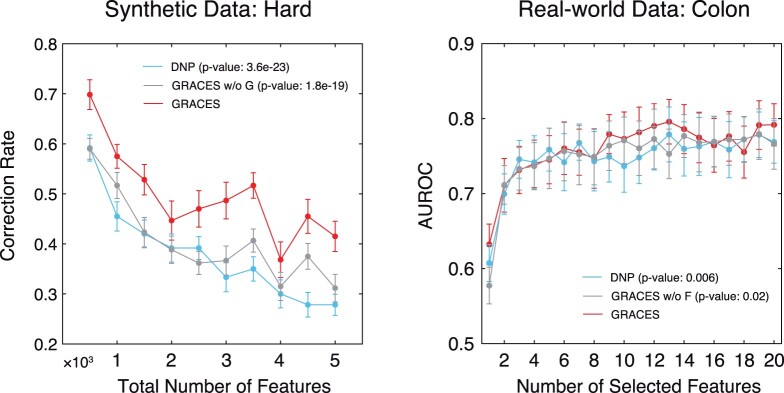
Ablation study on the two overfitting-reducing techniques. Left: average test correction rate of DNP, GRACES w/o G (GRACES without introduction of Gaussian noises), and GRACES with respect to the total number of features for the hard synthetic dataset. Right: average test AUROC of DNP, GRACES w/o F (GRACES without F-correction), and GRACES with respect to the number of selected features for the Colon dataset. Error bars indicate standard error mean, and *P*-values were computed using the one-sample paired t-test between GRACES and the other two

Both the synthetic and real-world datasets demonstrate compelling evidence that GRACES can achieve a superb and stable performance on HDLSS datasets. Notably, the two new overfitting-reducing techniques, i.e. introduction of Gaussian noises and F-correction, play critical roles in GRACES. We performed an ablation study to demonstrate the effectiveness of the two overfitting-reducing techniques. We tested the former on the same synthetic dataset with the hard mode and the latter on the Colon dataset, respectively. The results are shown in Fig. 5, where the performance of GRACES significantly deteriorates without introducing Gaussian noises (left) or F-correction (right). Nevertheless, even without using the two overfitting-reducing techniques, GRACES is still slightly better than the second-best method DNP in both the cases.

Next, we discuss two drawbacks of GRACES. First, according to the experiment on the synthetic datasets, although GRACES outperforms the other baseline methods, its performance also declines when the two classes are mixed intricately (i.e. the variable class_sep becomes small). Hence, GRACES might fail on data with highly nonlinear relations between features and labels. Second, GRACES is computationally inefficient. We computed the total computational time of each method for running the six biological datasets with selected features from 1 to 10, see Table 4. The ANOVA F-test is the most computationally efficient method among the seven methods. On the other hand, GRACES requires more computation resources in finding the optimal features due to its complex architecture. When the number of samples is small (e.g. Colon, Leukemia, ALLAML), the computational time of GRACES is still reasonable. However, when the number of samples becomes large (e.g. SMK_CAN_187), the computational time increases drastically. Therefore, GRACES is only applicable for HDLSS data and cannot handle normal feature selection tasks with large-sample sizes.

**Table 4:** Total computational time with selected features from 1 to 10 (in s) of each method for the six biological datasets.^a^

Dataset	Colon	Leukemia	ALLAML	GLI_85	Prost._GE	SMK._187
F-test	0.03	0.08	0.24	0.73	0.20	1.30
LR Lasso	0.16	0.57	0.66	1.74	0.85	3.81
HSIC Lasso	4.19	7.42	7.48	16.58	7.59	27.64
RF	0.52	0.63	0.88	1.62	0.90	3.60
CancelOut	1.37	5.14	5.21	12.84	5.60	26.03
DNP	5.05	5.53	5.57	8.99	6.16	13.59
GRACES	4.93	12.27	12.02	33.66	16.29	128.37

a We used the default hyperparameters of each method to obtain the computational time.

## 6 Conclusion

In this article, we proposed a deep learning-based method GRACES to perform feature selection on HDLSS data. By utilizing GCN along with different overfitting-reducing strategies including multiple dropouts, introduction of Gaussian noises, and F-correction, GRACES achieves a superior performance on both the synthetic and real-world HDLSS datasets compared to other classical feature selection methods. GRACES can be applied to many other types of biological datasets that suffer from the HDLSS problem. It will be useful to investigate more sophisticated network architecture to learn the low-dimensional representations of data. For example, hypergraph convolutional network (Feng et al. 2019; Bai et al. 2021; Chen and Liu 2022), generalized from GCN, is able to exploit higher-order associations among samples, which might result in a more accurate representation for each sample. Further, more overfitting-reducing techniques such as normalization can be considered.

## Supplementary Material

btad135_Supplementary_Data

## Data Availability

The data underlying this article are available in https://jundongl.github.io/scikit-feature/datasets.html.
