# Supplementary material for: Graph convolutional network-based feature selection for high-dimensional and low-sample size data
Source: Bioinformatics. 2023 Apr 21;39(4):btad135. doi: 10.1093/bioinformatics/btad135 (PMC10126323; doi:10.1093/bioinformatics/btad135)
Supplement: btad135_Supplementary_Data [file btad135_supplementary_data.pdf]

Graph Convolutional Network-based Feature Selection for High-dimensional and Low-sample Size Data

Can Chen[1], Scott T. Weiss[1], and Yang-Yu Liu[1,2,*]

[1]Channing Division of Network Medicine, Department of Medicine, Brigham and Women's Hospital and Harvard Medical School, Boston, MA 02115, USA

[2]Center for Artificial Intelligence and Modeling, the Carl R. Woese Institute of Genomic Biology, University of Illinois at Urbana-Champaign, Champaign, IL 61820, USA

[*]To whom correspondence should be addressed (yyl@channing.harvard.edu).

Supplementary Figures

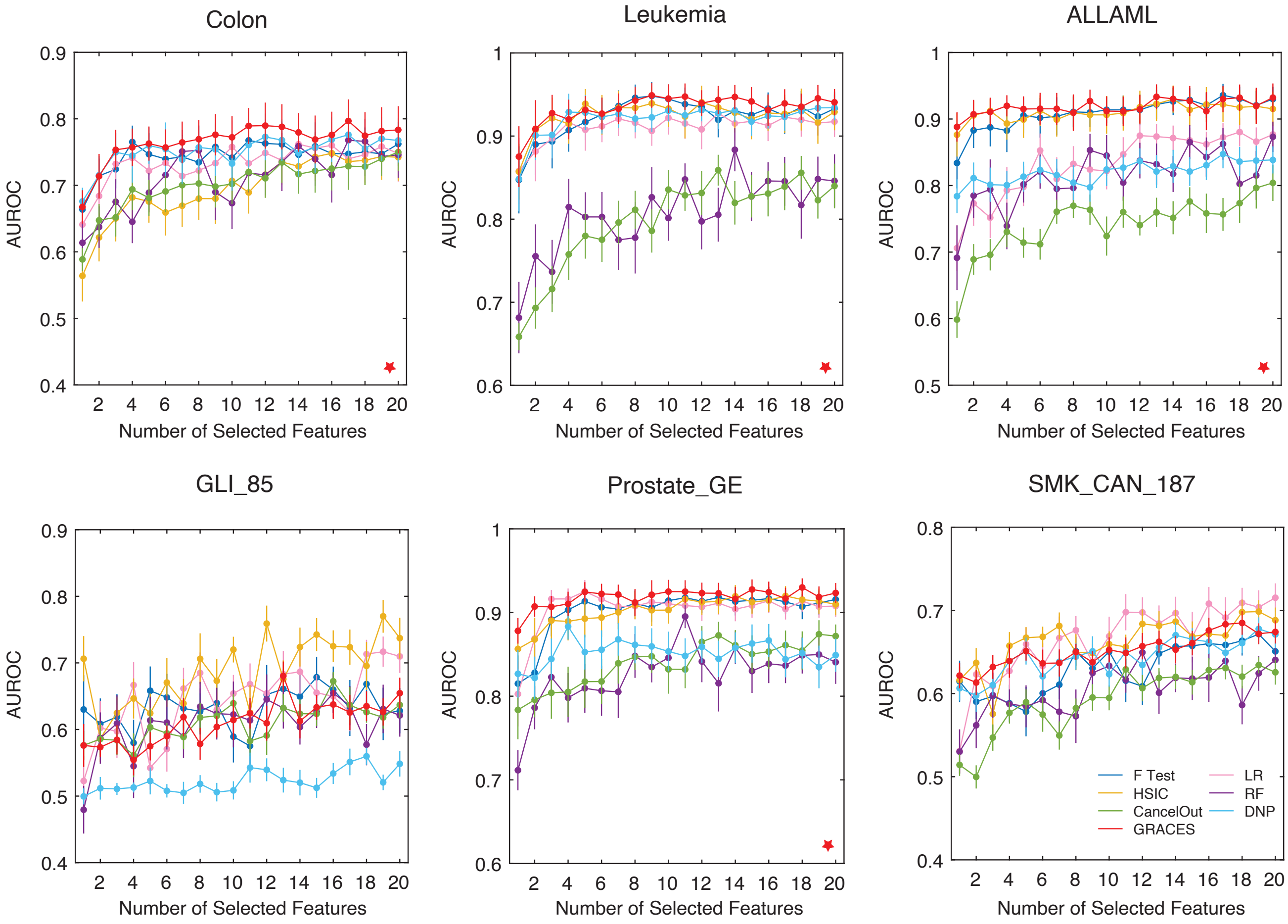

Fig. S1: Real-world datasets. Average test AUROC with respect to the number of selected features for each dataset with the MLP classifier. Error bars indicate standard error mean, and red stars indicate statistical significance compared to the second-best method (p-value < 0.05, one-sample paired t-test on the total 400 AUROC scores).

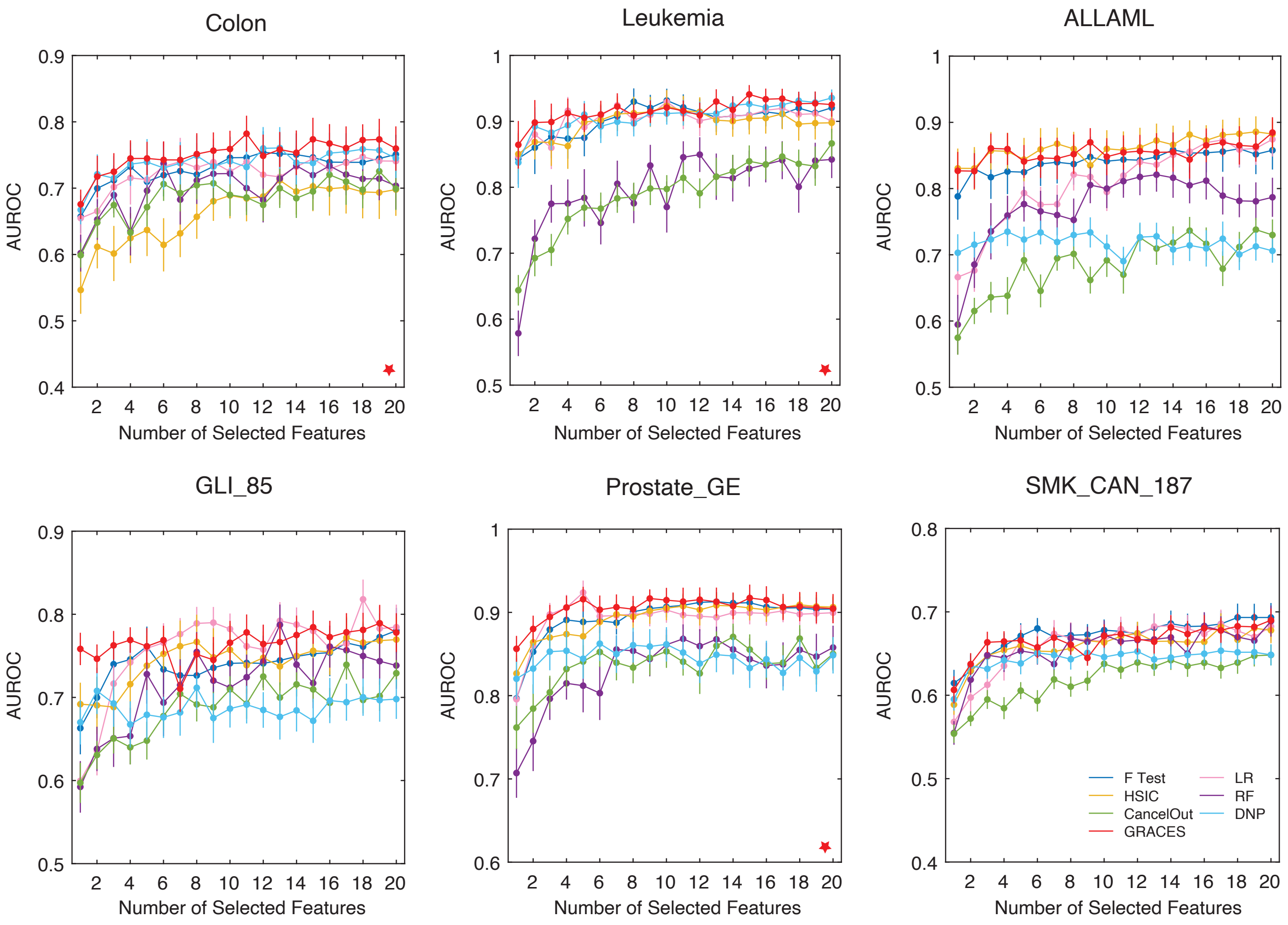

Fig. S2: Real-world datasets. Average test AUROC with respect to the number of selected features for each dataset with the k-nearest neighbors classifier. Error bars indicate standard error mean, and red stars indicate statistical significance compared to the second-best method (p-value < 0.05, one-sample paired t-test on the total 400 AUROC scores).
